# SR-BI Mediated Transcytosis of HDL in Brain Microvascular Endothelial Cells Is Independent of Caveolin, Clathrin, and PDZK1

**DOI:** 10.3389/fphys.2017.00841

**Published:** 2017-10-30

**Authors:** Karen Y. Fung, Changsen Wang, Steffen Nyegaard, Bryan Heit, Gregory D. Fairn, Warren L. Lee

**Affiliations:** ^1^Department of Biochemistry, University of Toronto, Toronto, ON, Canada; ^2^Keenan Research Center for Biomedical Science, St. Michael's Hospital, Toronto, ON, Canada; ^3^Program in Cell Biology, Hospital for Sick Children, Toronto, ON, Canada; ^4^Department of Microbiology and Immunology, Centre for Human Immunology, University of Western Ontario, London, ON, Canada; ^5^Department of Surgery, University of Toronto, ON, Canada; ^6^Departments of Medicine and Laboratory Medicine and Pathobiology, University of Toronto, ON, Canada

**Keywords:** HDL transcytosis, Blood-brain barrier, Endothelial cells, SR-BI, Endocytosis, Endothelial nitric oxide synthase, clathrin, caveolin

## Abstract

The vascular endothelium supplying the brain exhibits very low paracellular and transcellular permeability and is a major constituent of the blood-brain barrier. High-density lipoprotein (HDL) crosses the blood-brain barrier by transcytosis, but technical limitations have made it difficult to elucidate its regulation. Using a combination of spinning-disc confocal and total internal reflection fluorescence microscopy, we examined the uptake and transcytosis of HDL by human primary brain microvascular endothelial cell monolayers. Using these approaches, we report that HDL internalization requires dynamin but not clathrin heavy chain and that its internalization and transcytosis are saturable. Internalized HDL partially co-localized with the scavenger receptor BI (SR-BI) and knockdown of SR-BI significantly attenuated HDL internalization. However, we observed that the adaptor protein PDZK1—which is critical to HDL-SR-BI signaling in other tissues—is not required for HDL uptake in these cells. Additionally, while these cells express caveolin, the abundance of caveolae in this tissue is negligible and we find that SR-BI and caveolin do not co-fractionate. Furthermore, direct silencing of caveolin-1 had no impact on the uptake of HDL. Finally, inhibition of endothelial nitric oxide synthase increased HDL internalization while increasing nitric oxide levels had no impact. Together, these data indicate that SR-BI-mediated transcytosis in brain microvascular endothelial cells is distinct from uptake and signaling pathways described for this receptor in other cell types.

## Introduction

The cerebral circulation is characterized by a high degree of impermeability, an essential feature that protects the brain from fluctuations in ion concentrations and exposure to circulating toxins. The blood-brain-barrier (BBB) consists in large part of a continuous monolayer of endothelial cells joined by intercellular tight junctions that effectively occlude the intercellular space, reducing paracellular permeability. Additionally, brain endothelial cells also exhibit a low rate of endocytic activity relative to endothelium from other tissue beds which has been ascribed to low numbers of caveolae and intracellular vesicles (Reese and Karnovsky, 1967; De Bock et al., 2016). Recent studies have revealed that the expression of the major facilitator superfamily domain-containing protein 2 (Mfsd2a), which acts as a sodium-dependent lysophosphatidylcholine symporter, is the reason why caveola formation is largely absent in brain microvascular endothelial cells (Ben-Zvi et al., 2014; Andreone et al., 2017).

At the same time, the impermeability of the blood-brain barrier is not absolute, allowing the regulated transport of various molecules such as glucose, amino acids and electrolytes and also larger molecules such as low-density and high-density lipoproteins (LDL; Dehouck et al., 1997 and HDL; Vitali et al., 2014). Movement of LDL and HDL across the BBB is thought to occur by transcytosis, in which the lipoprotein is internalized at the luminal surface by the endothelium, trafficked through the individual endothelial cell and then ultimately exocytosed at the basal membrane (Mehta and Malik, 2006; Armstrong et al., 2015). Elucidating the mechanisms of HDL transcytosis across the blood-brain barrier may be significant pathologically as its constituent apolipoprotein ApoA1 has been demonstrated to confer a protective effect against Alzheimer disease (AD). In AD, aggregation and deposition of amyloid beta (aβ) protein disrupt neuronal communication and induces neuronal cell death; *in vitro* data suggests that ApoA1 binds and prevents the aggregation of aβ, thus reducing its toxicity (Koldamova et al., 2001). Furthermore, in mouse models, ApoA1 deficiency exacerbated cognitive defects (Lefterov et al., 2010) while its overexpression was protective. Thus, manipulation of HDL transcytosis to (or intending to) increase delivery of ApoA1 has been suggested as a potential therapeutic approach for AD. More broadly, enhancement of endothelial transcytosis (e.g. of HDL-like synthetic particles) across the blood-brain barrier has been proposed as a drug delivery tool to the brain (Balazs et al., 2004). This, however, would require an exquisite understanding of its regulation.

To date, most studies investigating transcytosis of HDL have used non-vascular tissues (e.g., hepatocytes, Silver et al., 2001) or endothelial cells from the systemic circulation such as the umbilical vein (Fruhwürth et al., 2013) or the aorta (Rohrer et al., 2009). In the systemic circulation, binding of HDL to scavenger receptor BI (SR-BI) culminates in phosphorylation of endothelial nitric oxide synthase (eNOS) and nitric oxide (NO) production that is dependent on the adaptor protein PDZK1 (Zhu et al., 2008). As endothelial cells are quite heterogeneous depending on the tissue bed (Aird, 2007a; Azizi et al., 2015) and since the blood-brain barrier has unique structural and functional properties, whether this canonical signaling pathway is required for HDL transcytosis across the blood-brain barrier is unknown.

Finally, due to longstanding technical limitations—namely the difficulty distinguishing paracellular diffusion from transcytosis in cultured endothelial cells—relatively little is known about the molecular mechanisms of receptor-mediated transcytosis across the blood-brain barrier, including HDL transcytosis. In particular, endothelial transcytosis has traditionally been studied using electron microscopy (EM; Palade, 1961) or by seeding cells on Boyden chambers (transwells). However, EM studies are largely descriptive while the study of transcytosis using transwells is easily confounded by the development of paracellular gaps in response to pharmacological inhibitors or molecular manipulation (Armstrong et al., 2012). Thus, a novel approach to the study of endothelial transcytosis is needed to facilitate the delineation of its mechanisms.

In this study, we use high-resolution fluorescence microscopy to observe the internalization of HDL in primary human brain microvascular endothelial cells. We describe a single-cell assay based on total internal reflection fluorescence (TIRF) microscopy to elucidate the proximal signaling events required for HDL transcytosis. Using this approach, we now report that HDL internalization by brain endothelial cells is mediated at least in part by SR-BI but does not require either the adaptor protein PDZK1 or endothelial nitric oxide synthase activation.

## Materials and methods

### Cell culture

Primary human cerebral cortex microvascular endothelial cells (HCCMECs, Cell Systems: ACBRI 376) were cultured in endothelial cell medium (ScienCell Research Laboratories) and grown in 37°C at 5% CO_2_. Human aortic endothelial cells (HAECs, passage 4, Lonza: CC-2535) were cultured in EGM-2^TM^ BulletKit^TM^ medium (Lonza, Switzerland) under the same environmental conditions. The medium was changed every 2–3 days of culture. Unless stated otherwise, experiments were performed at least 2 days after subculture between passages 7–9.

### Labeling HDL

Commercially purchased human HDL (Kalen Biomedical LLC) was labeled with Alexa Fluor 568 NHS Ester (ThermoFisher Scientific) following the manufacturer's protocol. Following conjugation, the reaction was dialyzed against 3x1L sterile 1x PBS over 3 days at 4°C using Slide-A-Lyzer^TM^ Dialysis Cassettes, 10K MWCO, 0.5 mL (ThermoFisher Scientific) where dialysis buffer was changed after each day. The efficiency of Alexa Fluor labeling was verified by running dialyzed Alexa Fluor labeled HDL (AF568-HDL) on an SDS-PAGE to compare the amount of Alexa Fluor labeled ApoA1 (approximately 25 kDa) to free Alexa Fluor. Additionally, to ensure the HDL particle remained intact after Alexa Fluor labeling, the size of AF568-HDL was compared with unlabeled HDL by running on an agarose gel followed by lipid staining with Sudan Black B (Sigma-Aldrich).

### Recombinant ApoA1 synthesis

A codon optimized cDNA encoding the human ApoA1 was synthesized with flanking restriction enzymes for subcloning by Integrated DNA Technologies DNA (Coralville, Iowa). The open reading frame was subcloned into the pET28b vector (Novagen) This recombinant ApoA1 was expressed in endotoxin-deficient ClearColi® BL21 (DE3) cells (Lucigen) and purified as previously described (Ryan et al., 2003), using a Talon resin (Clontech) (see Supplemental Figure 1).

### Internalization assay

#### Internalization assay

HCCMECs were grown to confluency on 0.1% gelatin (Sigma-Aldrich)-coated 18 mm coverslips. Next, 10 μg/mL of DiI (1,1′-dioctadecyl- 3,3,3′,3′-tetramethylindocarbocyanine perchlorate) - HDL (Alfa Aesar) or 20 μg/mL of AF568-HDL in RPMI supplemented with HEPES (HPMI; Gibco) were incubated with HCCMECs for 10 min at 37°C. Labeled HDL was aspirated followed by two washes with PBS containing calcium and magnesium (PBS+). HCCMECs were then fixed for 20 min in 4% PFA (Electron Microscopy Sciences) at room temperature followed by treatment with 0.15% glycine in PBS for at least 15 min at room temperature. Coverslips were mounted in mounting media (Dako) supplemented with 1 ug/mL DAPI (Sigma-Aldrich). Coverslips were imaged using the spinning disk microscope (Olympus IX81, at 63x objective, numerical aperture 1.35) at a z-stack interval of 0.3 μm with settings kept constant between conditions.

#### Inhibitor pre-treatments

For competition with unlabeled HDL, a 40-fold excess of HDL (by mass) was added along with 10 μg/mL DiI-HDL or 20 μg/mL AF568-HDL. To inhibit dynamin and caveolae, HCCMECs were treated with 30 μM Dyngo 4a (Abcam, Cambridge, MA) or 50 μg/mL Nystatin (Bioshop Canada, Burlington, ON) in HPMI at 37°C for 30 min prior to the addition of labeled HDL. Nitric oxide donor spermine NONOate (40 μM) (Abcam, Cambridge, MA) or endothelial nitric oxide synthase inhibitor L-NNA (1.6 μM) (Sigma-Aldrich) made up in serum-free media were added to the cells 3 h prior to the addition of labeled HDL. For all pre-treatments, the inhibitors used were re-added during the 10 min internalization step.

#### SR-BI single-chain variable fragment

Conformation specific, anti-SR-BI single-chain variable fragment (scFv) was a kind gift from Prof. Alfredo Nicosia (Catanese et al., 2007). To avoid the unwanted crosslinking of SR-BI, a scFv was generated from the IgG sequence and validated to still be fully functional in competition with AF568-HDL bound to GFP-SR-BI expressing CHO cells. Similarly, the binding of labeled scFv could be inhibited by competition with 10-fold excess of unlabeled scFv. For visualization the scFv was labeled with Atto488 succinimidyl ester (Atto-tec, Siegen, GmbH) using the same protocol as above for AF568-HDL. The resulting scFv was labeled in a 1:1–1:2 stoichiometry as verified by bleaching similar to Jaqaman et al. (2011).

Live cell microscopy was used to assess the degree of HDL internalization inhibition by the SR-BI scFv. Briefly, HCCMECs were pre-treated with 10 μg/mL of the anti-SR-BI for 5 min at 4°C.Next, 20 μg/mL of the AF568-HDL was added and then incubated for an additional 20 min at 4°C. HCCMECs were washed twice with PBS(+) and allowed to internalize for 10 min at 37°C after which images were taken using the spinning disk microscope (Olympus IX81, at 63x objective, numerical aperture 1.35) at a z-stack interval of 0.3 μm.

#### Quantification

The amount of HDL internalized was calculated by the sum of the integrated density of HDL signal across all z-slices using ImageJ software (NIH). This was normalized to the total number of nuclei observed for each treatment. This value was subtracted by the normalized integrated density of HDL in images taken where no labeled HDL was added.

### Total internal reflection fluorescence transcytosis assay

Total internal reflection fluorescence (TIRF) microscopy videos were taken of HCCMECs seeded on 25 mm glass coverslips in a cell chamber on the Olympus cell TIRF Motorized Multicolor TIRF module mounted on an Olympus 1X81 microscope (Olympus, Hamburg, Germany). Samples were imaged using a 150x/1.45 NA objective with 561 nm excitation lasers and Volocity acquisition software. The penetration depth or the distance into the basal end of the cells that are excited was set at 110 nm. For each cell, 150 TIRF images were taken at a frame rate of 6.67 per second for a constant duration of approximately 22 s. At least 8 randomly selected fields were imaged in each experimental replicate. Unless stated otherwise 2.5 μg/mL AF568-HDL and nuclear stain NucBlue® Live ReadyProbes® (Molecular Probes, Oregon, United States) were added and incubated 10 min at 4°C. Cells were washed twice with PBS(+) after which HCCMECs were returned to fresh warm HPMI; the cell chamber was placed on a heated stage (37°C) stage and after 2 min images were taken. For competition with unlabeled HDL, cells were pre-chilled for 5 min at 4°C. 40x excess of unlabeled HDL was added with 2.5 μg/mL of the AF568-HDL and allowed to bind for 10 min at 4°C after which the coverslip was washed. Dyngo4a was added to the cells for 30 min at 37°C prior to HDL binding in the cold; Dyngo4a was left in the binding and imaging step. To inhibit SNARE-mediated exocytosis, 2.5 μg/mL AF568-HDL was allowed to bind for 10 min at 4°C after which the coverslip was washed and 2 μM N-ethylmaleimide (NEM) was added only during the imaging step.

Blinded and automated quantification of the transcytotic events was performed using a tracking algorithm for MATLAB as previously reported (Armstrong et al., 2015; Azizi et al., 2015). Briefly, the scripts correct the image for noise and local background using a Gaussian filter. Then putative vesicles are identified based on size (9–36 pixels^2^, XY dimension, 73.5 nm/pixel), aspect ratio (>0.2; the ratio of the minor axis to the major axis) and intensity (threshold of 10% above mean image intensity). The tracking algorithm then tracks each moving vesicle based on a maximum-probability assessment of how closely those potential tracks resemble free and super-diffusive Brownian diffusion. The resulting tracks are analyzed for the duration of the vesicle being stationary in the TIRF field (vesicle docking), the speed of vesicular movement and the degrees to which the particles' movements deviates from free Brownian diffusion (γ). Vesicles undergoing fusion with the plasma membrane are identified as those having a γ significantly less than that of an equivalent model population undergoing Brownian diffusion, typically 0 < γ < 0.873, and which undergo a decrease in fluorescence signal over the last two time points of their tracks equivalent to a drop of at least 2.5 standard deviations of vesicular intensity over the entire period the vesicle has been tracked.

### Immunofluorescence staining and co-localization assay

#### Junctional markers

To probe for junctional protein VE-Cadherin and ZO-1, a confluent monolayer of HCCMECs was fixed with cold methanol in −20°C for 15 min. After washing with PBS, HCCMECs were blocked with 5% BSA for at least 30 min. After washing twice with PBS, HCCMECs were incubated with primary antibodies against VE-Cadherin (Santa Cruz Biotechnology) and ZO-1 (Invitrogen) made up in 2.5% BSA at a concentration of approximately 5 μg/ml for 1 h at room temperature. HCCMECs were washed twice with PBS and incubated with their corresponding Alexa Fluor-conjugated secondary antibodies (Jackson ImmunoResearch) at 1.5 μg/ml in PBS. Cells were washed twice with PBS and mounted in mounting media supplemented with 1 μg/mL DAPI. Images were acquired with a spinning disk microscope (Olympus IX81, at 20x objective, numerical aperture 1.35) at a z-stack interval of 0.3 μm.

#### HDL and SR-BI co-localization

To assess HDL and SRBI co-localization, 20 μg/mL AF568-HDL and 1 μg/mL Atto488-anti-SR-BI scFv were incubated with HCCMECs for 10 min at 4°C to allow for membrane binding. Cells were then washed with PBS(+) and allowed to internalize for 5 min at 37°C after which images were taken using the spinning disk microscope (Olympus IX81, at 63x objective) at a z-stack interval of 0.3 μm. Co-localization was calculated using Volocity 6.0.1 and reported using Manders' correlation coefficient for the amount of HDL co-localizing with SR-BI. Regions of interest (ROI) were used to include individual cells when Manders was calculated. To evaluate significance we calculated the Manders after rotating the HDL ROI 180° and compared to the original SR-BI image.

#### Tracking the polarized transport of HDL

For z-axis imaging of the fate of HDL over time, HCCMECs were incubated with 20 μg/mL AF568-HDL in HPMI at 4°C for 10 min for membrane binding. At time = 0, 20 μg/mL fluorescein labeled elderberry bark lectin (Vector Labs, Burlington, ON, Canada) was added to the media during membrane binding to identify the apical plasma membrane for a subset of coverslips of HCCMECs. These were then rinsed twice with PBS (+) and fixed in 4% PFA for 30 min at 4°C.The remaining coverslips of HCCMECs were returned to warm HPMI and placed in the 37°C incubator for the indicated times. Over the last 5 min of incubation, 20 μg/mL of the fluorescein-labeled lectin was added to the media after which HCCMECs were washed twice with PBS(+) and fixed. Fixation was stopped with 0.15% glycine in PBS for at least 15 min at room temperature and then mounted on slides using mounting media supplemented with DAPI. Images were acquired with a spinning disk microscope (Olympus IX81, at 63x objective) at a z-stack interval of 0.3 μm.

The distribution of HDL was quantified using ImageJ (NIH) by measuring total cellular HDL and the signal proximal to the apical membrane as marked by the fluorescent lectin of the xy slice in the middle of the cell. The percent of the HDL proximal to the apical membrane was calculated by dividing the integrated intensity of the proximal pixels by the total integrated pixel intensity throughout the cell. The intracellular HDL was determined by subtracting the apical HDL from the total HDL signal. This corrected value was then used to generate a percentage by dividing the intracellular HDL signal by the total HDL signal.

### Isolation of detergent resistant membranes

Confluent endothelial cells in a 6 cm dish were washed twice with cold PBS(+) and then scraped into 0.5 mL of cold PBS(+). The cells were collected by centrifugation for 5 min at 2,000 rpm, 4°C. Next, cells were resuspended and lysed in 100 μL of TN buffer (25 mM Tris pH 7.5, 150 mM NaCl, 10% Sucrose, 1% TX-100, 1 mM DTT, supplemented with protease inhibitors) for 30 min with shaking. The lysate was transferred to a 1 mL Wheaton 33 low extractable borosilicate glass mortar of the tapered tissue grinder with a clearance of 0.1–0.15 mm (Wheaton, USA–item #: 358133) along with 700 μL of 60% Optiprep™ (Axis-Shield, Oslo Norway). This solution was homogenized with 20–25 strokes with the steel rod attached PTFE pestle of the tissue grinder on ice and then transferred to the bottom of a ½ × 2 inch ultracentrifuge tube (Beckman Coulter, USA). Equal volumes of 40, 30, and 20% Optiprep™ (in TN buffer) were then layered on top in decreasing percentage. This was then subjected to centrifugation at 28,000 rpm for 18 h at 4°C in a Beckman Coulter L8-70 M Ultracentrifuge using a SW55Ti rotor. A total of 8 fractions (2 fractions per 10% change in Optiprep™) were collected from this density gradient and subsequently the protein was concentrated using methanol:chloroform precipitation. One fraction from each percentage Optiprep™ was then analyzed through western blot. For western blots, primary antibodies against the VE-Cadherin (Santa Cruz: sc-6458), cav-1 (Santa Cruz: sc-894), and SR-B1 (Novus Biological: NB400-104) were incubated with the membrane overnight at 4°C with shaking. Horseradish peroxidase-conjugated secondary against the appropriate species was then added for 1 h at room temperature and the blot was then developed.

### Measuring nitric oxide production with DAF-FM

To measure nitric oxide production, 5.2 μM 4-Amino-5-Methylamino-2′,7′-Difluorofluorescein (DAF-FM) Diacetate (Molecular Probes, Oregon, United States) made up in HBSS was added to HCCMECs and incubated at 37°C for 30 min. DAF-FM was aspirated and the cells were washed once with HBSS and resuspended with HBSS supplemented with NucBlue® Live ReadyProbes®. The fluorescent signal was captured using the ImageXpress Widefield High-Content Analysis System (Molecular Devices, California, United States) at 10x magnification in the DAPI and FITC channel. The average integrated intensity per cell was measured and calculated by the MetaXpress software (Molecular Devices).

### Transfection

#### siRNA transfection

Depletion of clathrin heavy chain and SR-BI was accomplished by transfection of siRNA with HiPerfect transfection reagent (Qiagen) as per the manufacturer's protocol. Cells were topped up with ECM 4–6 h post transfection and 24 h later, the medium was changed. Experiments were performed 48 h post transfection. Caveolin-1 was depleted using the Neon Transfection System (Invitrogen) as per the manufacturer's protocol under the following settings: one pulse at 1,650 V for 30 ms. Experiments were performed 48 h post transfection without the need for daily media replacement. All siRNAs were purchased from Qiagen (Valencia, CA, USA): the negative targeting control (NTC) siRNA (Cat. No. 1027310), SR-BI (Cat. No. SI02777201), clathrin heavy chain (Cat. No. SI00299880), Cav-1 (Cat. No. GS857).

#### Plasmid transfection

Overexpression of fusion proteins was carried out through electroporation using the BTX 830 system (BTX, Holliston, MA) with the following settings: 200 V, 1 pulse for the duration of 45 ms. Before electroporation, HCCEMCs were resuspended in OPTIMEM (Gibco-supplemented with 10% FBS) at a concentration of 3 × 10^6^ cells/mL and chilled on ice for 10 min. After electroporation, HCCMECs were returned to wells containing half the volume of ECM, this was topped off with additional ECM 4 h post electroporation. Following 24 h of incubation the medium was changed and experiments were performed 48 h post electroporation.

#### Assessing transfection and silencing

The degree of knockdown or overexpression was assessed through either immunofluorescence or Western blot analysis as indicated. For western blots, primary antibodies against the clathrin heavy chain (Santa Cruz: sc-6579), cav-1 (Santa Cruz: sc-894), PDZK1 (Santa Cruz: sc-100337), α-actinin (Cell Signaling: #3134), GAPDH (Santa Cruz: sc-25778) and β-actin (Santa Cruz: sc-47778) were incubated with the membrane overnight at 4°C with shaking. Horseradish peroxidase-conjugated secondary against the appropriate species was then added for 1 h at room temperature and the blot was then developed. Depletion of caveolin and clathrin was assessed through Western blot by lysing the cells in SDS Lysis Buffer 2 days after transfection. The degree of expression was measured by ImageJ or Image Studio Lite (LI-COR) and normalized to the loading controls. This was then expressed as the proportion compared to the control siRNA. To detect SR-BI depletion, NTC or SR-BI siRNA was co-transfected using HiPerfect with a mCherry construct (molar ratio 100:1 siRNA to mCherry) to identify cells that have taken up the siRNA. 48 h post transfection, endogenous SR-BI was labeled in live cells using 1 μg/mL Atto488-anti-SR-BI nanobody in mCherry-positive HCCMECs using a spinning disk microscope (Olympus IX81, at 20x objective, numerical aperture 1.35) at a z-stack interval of 0.3 μm. In the SR-B1 siRNA transfected coverslip, cells that were negative for mCherry expression had SR-BI staining comparable to untreated controls. The mCherry-positive cells (also took up SR-BI siRNA) showed essential no endogenous SR-BI immunofluorescent signal.

### Statistics

Statistical analysis was performed using the GraphPad Prism software (GraphPad Prism 5.0; GraphPad Software Inc., La Jolla, CA, USA). Student's *t*-tests and one sample *t*-tests (GraphPad, La Jolla, CA, USA) were used to determine the significance of raw or normalized data (respectively). Data are presented as mean ± SEM. All experiments contained >10 technical replicates and were performed at least three times on different batches of cells. *Post-hoc* power analysis was performed using the Statistical Power Calculator (Decision Support Systems, Fort Worth, Texas) with a 5% alpha error level. All experiments had statistical power of 90-100%.

## Results

### Establishment of an *in vitro* primary human brain microvascular endothelial model

**Figure 1:**
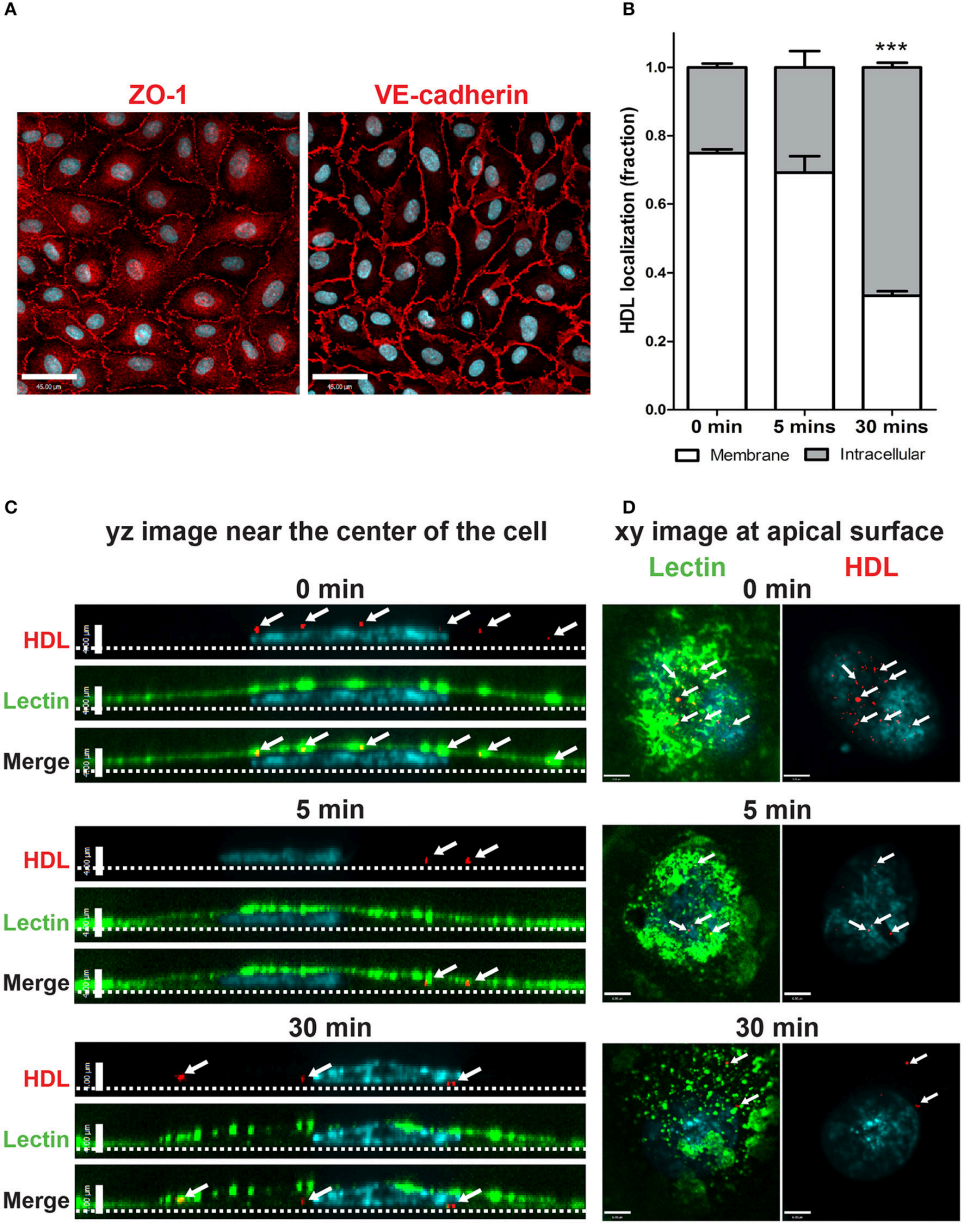
Establishment of an *in vitro* primary human brain microvascular endothelial model. **(A)** HCCMECs stained for tight junction protein ZO-1 (left) and adherens junction protein VE-cadherin (right). Scale Bars = 45 μm. **(B)** Quantification of pulse-chase of fluorescent-HDL. *n* = 3; about 15 cells were observed for each timepoint for each n. ^***^p < 0.0001 by Student's *t*-test for 0 vs. 30 min. **(C)** HDL was allowed to bind HCCMECs at 4°C for 10 min followed by rinsing to remove unbound HDL. Representative yz-axis images (scale bar = 4 μm) near the center of the cell. The apical membrane is labeled with lectin (green) while nuclei are stained with DAPI (blue). At time zero, HDL-containing vesicles (white arrows) can be seen at the apical cell surface; internalization is apparent after 5 min at 37°C. After 30 min, most of the HDL is internalized and appears near the basal membrane (dotted line). **(D)** Representative xy images (scale bar = 6 μm) of the apical membrane of HCCMECs are shown.

Primary human cerebral cortex microvascular endothelial cells (HCCMECs) were tested to see if their physiological properties are maintained when grown *in vitro*. They exhibited typical cobblestone morphology by phase contrast microscopy and formed monolayers with few gaps when grown to confluency (not shown). HCCMECs express junctional proteins VE-Cadherin and ZO-1 which enriched circumferentially at the plasma membrane where contacts are made with neighboring cells (Figure 1A). This suggests that HCCMECs are able to form cell-cell contacts essential for their barrier function.

The process of HDL transcytosis across endothelial cells *in vivo* occurs through a polarized manner from the apical to the basolateral end. This was tested in HCCMECs *in vitro* by labeling the apical membrane of HCCMECs on glass coverslips with elderberry bark lectin, (Armstrong et al., 2015) binding Alexa 568-labeled HDL (AF568-HDL) in the cold (4°C) and monitoring its intracellular localization over time. At time zero, most of the HDL (approximately 75%) was near the apical membrane whereas after 30 min, only 33% of total HDL remained near the apical membrane (*p* < 0.0001, *n* = 3); the remainder was intracellular and located at or approaching the basal membrane (Figures 1B–D). Together, these data suggest that HCCMEC maintain their polarized character *in vitro* and constitute a suitable model to study HDL transcytosis.

### HDL internalization requires dynamin and cholesterol but is clathrin and Cav-1 -independent

**Figure 2:**
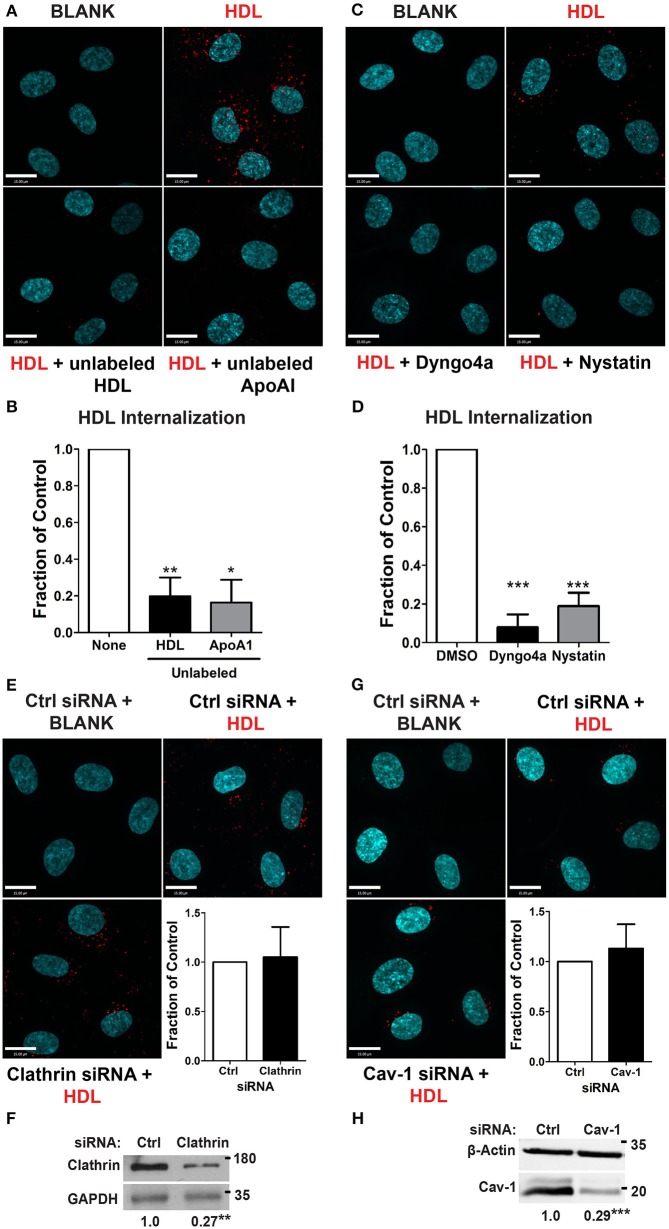
HDL internalization requires dynamin but not clathrin nor caveolin-1. **(A)** Internalization of Alexa 568-labeled HDL was significantly inhibited by 40-fold excess of unlabeled HDL, and 400 ug of recombinant ApoA1 protein. Quantification can be found in **(B)**; *n* = 6 for unlabeled HDL and *n* = 3 for recombinant unlabeled ApoA1, approximately 40 cells were observed per condition for each replicate. **(C)** 30 min pre-treatment with inhibitors of dynamin (Dyngo4a) and cholesterol (Nystatin) significantly decreased HDL internalization. Quantification can be found in **(D)**; *n* = 6; approximately 32 cells were observed for each treatment for each n. **(E)** Depletion of clathrin heavy chain by siRNA had no effect on HDL internalization. *n* = 4; approximately 25 cells were observed for non-targeting control (NTC) siRNA and clathrin siRNA for each n. Scale Bars = 15 μm. **(F)** Clathrin depletion as verified through western blot which showed a fold change of approximately 0.27 ± 0.065 of clathrin heavy chain protein expression compared to the control siRNA. **(G)** Depletion of Cav-1 by siRNA also had no effect on HDL internalization. *n* = 4; approximately 40 cells were observed for non-targeting control (NTC) siRNA and clathrin siRNA for each n. Scale Bars = 15 μm. **(H)** Cav-1 depletion as verified through western blot which showed a fold change of approximately 0.29 ± 0.027 of Cav-1 protein expression compared to the control siRNA. ^*^p < 0.05; ^**^p < 0.005; ^***^p < 0.0001.

We began by focusing on the initial stage of HDL transcytosis, namely its internalization. DiI (1,1′-dioctadecyl- 3,3,3′,3′-tetramethylindocarbocyanine perchlorate) labeled HDL (DiI-HDL) was incubated with HCCMECs for 10 min at 37°C, leading to the appearance of intracellular red punctae (Figure 2A). The addition of 40-fold excess unlabeled HDL largely abrogated the fluorescent signal, consistent with competition for a cell-surface receptor. Similarly, the addition of 40-fold excess by mass of recombinant ApoA1 also inhibited HDL internalization (Figures 2A,B, 83% decrease compared to control). Accordingly, pre-treatment with the dynamin GTPase inhibitor Dyngo4a almost entirely blocked DiI-HDL internalization (Figures 2C,D, 92% decrease compared to labeled HDL alone). As dynamin is necessary for both clathrin- and caveola-mediated endocytosis (Oh et al., 1998), as well as other modes of internalization (Conner and Schmid, 2003), we depleted HCCMECs of clathrin heavy chain by siRNA transfection (approximately 70% depletion by western blot, Figure 2F); this led to no significant change in HDL internalization (Figure 2E). Recent studies have demonstrated that the presence of Mfsd2a largely abrogates caveola formation in brain microvascular endothelial cells (Andreone et al., 2017). To determine if the low-levels of caveolae could still be important in the uptake process we decided to test whether HDL is internalized through caveola-mediated endocytosis. In these experiments significant Cav-1 depletion (≈71% depletion by Western blot, Figure 2H) had no impact on HDL internalization (Figure 2G). Curiously, treatment of HCCMECs with the cholesterol-binding compound nystatin significantly decreased the amount of internalized HDL (reduction of about 81%; see Figures 2C,D). Thus, HDL internalization uses a dynamin- and cholesterol-dependent pathway distinct from clathrin and caveola.

### A single-cell assay to quantify HDL transcytosis by HCCMECs

**Figure 3:**
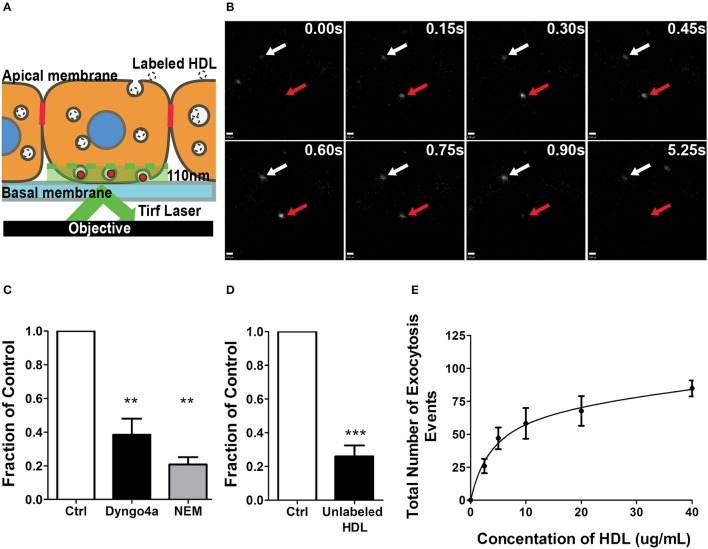
A single-cell assay to quantify HDL transcytosis. **(A)** Schematic of the TIRF assay. Fluorescently labeled HDL was added to apical surface of a confluent monolayer of HCCMECs and is detected by TIRF microscopy as it enters the field of illumination at the bottom of the cell. **(B)** Still images from representative TIRF videos. White arrows point to vesicles that do not fuse while the red arrow shows an HDL containing vesicle disappearing as it is undergoing exocytosis. Scale Bars = 0.60 μm. **(C)** 30 min pretreatment with Dyngo4a or the addition of N-ethylmaleimide (NEM) after allowing HDL to bind to the apical membrane both decreased the number of HDL transcytosis events. *n* = 6 for Dyngo4a and *n* = 3 for NEM; approximately 8 cells were observed for each n. **(D)** 40-fold excess unlabeled HDL largely abrogated HDL transcytosis measured by TIRF. *n* = 4; approximately 8 cells were observed for each n. **(E)** HDL transcytosis is saturable. *n* = 4; approximately 8 cells were observed for each n. ^**^p < 0.005, ^***^p < 0.0001 vs. control.

As alluded to earlier, relatively little is known about the molecular mechanisms of HDL transcytosis due to the limitations of both EM and transwell studies. In particular, transwells are often confounded by paracellular leak and poor transfection efficiency (particularly of primary cells), while EM studies are often descriptive rather than quantitative. To circumvent these issues, we developed a method of quantifying transcytosis by single endothelial cells in a confluent monolayer (Armstrong et al., 2015; Azizi et al., 2015) by detecting the exocytosis of internalized fluorescently-labeled HDL through total internal reflection fluorescence microscopy (TIRFM) (see Schematic, Figure 3A; Steyer et al., 1997). Quantification is performed in a blinded manner using an automated MATLAB script. The TIRF assay obviates issues such as low transfection efficiency since transcytosis can be examined in individual cells within the monolayer. Importantly, this assay avoids the edge-effects of cells seeded on a transwell and is not affected by paracellular leak since it focuses on individual cells in the confluent monolayer.

AF568-HDL was added to the apical surface of the endothelial monolayer and could be detected entering the evanescent field and undergoing exocytosis (Figure 3B). The addition of 40-fold excess unlabeled HDL significantly decreased the number of fusion events (Figure 3C), again suggesting competition for a receptor. Pre-treatment with Dyngo4A or preventing exocytosis by adding N-ethylmaleimide (NEM) - an inhibitor of SNARE complex disassembly and recycling (Söllner et al., 1993) - both significantly decreased HDL transcytosis to the basal membrane (Figure 3D). Finally, a dose-response curve indicated that HDL transcytosis was saturable (Figure 3E) consistent with it being a receptor-mediated uptake pathway.

### Scavenger receptor BI mediates HDL internalization in HCCMECs

Studies in aortic endothelial cells have implicated SR-BI in HDL transcytosis (Rohrer et al., 2009); we chose to focus our initial experiments on this receptor. SR-BI is a bifunctional protein with the ability to function as both a scavenger receptor as well as a channel allowing for the bi-directional movement of cholesterol between the plasma membrane and lipoproteins (Neculai et al., 2013). However, its function as an endocytic receptor is less well characterized (Fruhwürth et al., 2013) especially in the cerebral circulation.

**Figure 4:**
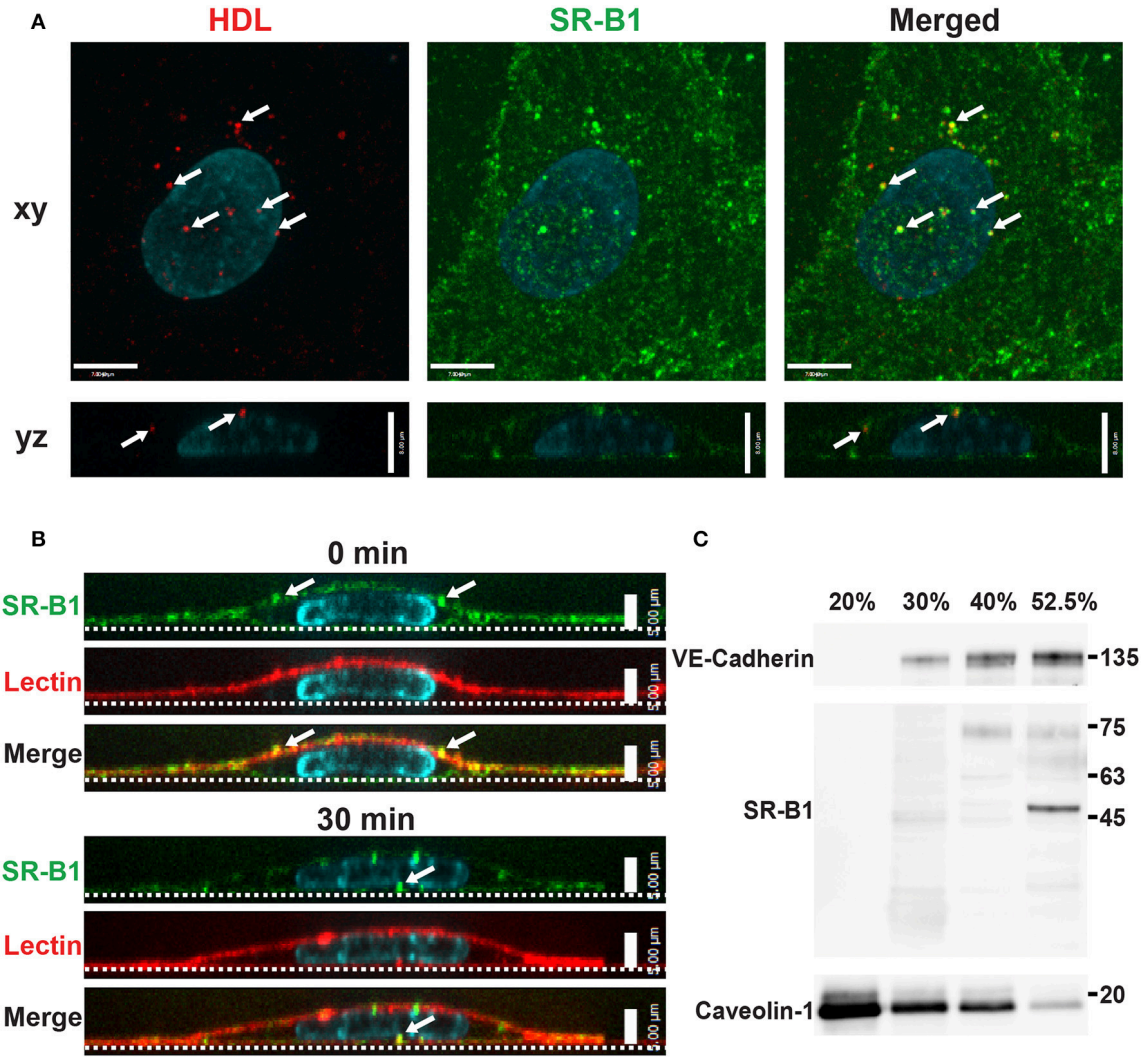
Scavenger Receptor BI co-localizes with HDL but does not reside in detergent-resistant membranes **(A)** Immunostaining of endogenous SR-BI (green) after addition of Alexa 568-HDL (white arrows) for 5 min at 37°C. White arrows in merged images indicate areas of colocalization; Manders coefficient of HDL to SR-BI = 0.247. *n* = 3; approximately 24 cells were observed for each n. Compared to colocalization observed by chance (i.e., HDL ROI rotated 180°). **(B)** Cell surface SR-BI (green) was labeled and cells were incubated at 37°C for 30 min. Images show internalization of the receptor over time (white arrows); the apical membrane is stained with lectin (red). **(C)** Density gradient fractionation of HCCMECs lysate revealed that endogenous SR-BI migrates with the VE-cadherin-containing subfractions (30–52.5% optiprep) and absent from fractions enriched for caveolin-1 (20% optiprep). Representative blot shown; *n* = 4 experiments.

To begin to investigate the participation of SR-BI in HDL internalization, the localization of SR-BI was determined by immunofluorescence. Endogenous SR-BI was enriched at the cell surface and on intracellular vesicles. Internalized AF568-HDL co-localized with endogenous SR-BI to the degree that was significantly higher than expected by chance (Manders Correlation Coefficient for HDL to SR-BI = 0.247; *p* < 0.0001, Figure 4A). The fact that co-localization is partial is perhaps unsurprising given that other receptors are known to bind HDL (Calvo et al., 1997, 1998). Additionally, cell-surface SR-BI was also observed to be internalized over time in HCCMECs (Figure 4B), mimicking the behavior of surface bound HDL (Figures 1B–D, section Cell Culture). Unexpectedly, in contrast to previous reports of SR-BI residing in lipid rafts in human microvascular endothelial cells (Uittenbogaard et al., 2000), this does not appear to be the case in human brain endothelial cells as SR-BI co-fractionated with the non-lipid raft plasma membrane marker VE-Cadherin (Figure 4C). This is likely due in part to the lack of caveolae in HCCMECs.

**Figure 5:**
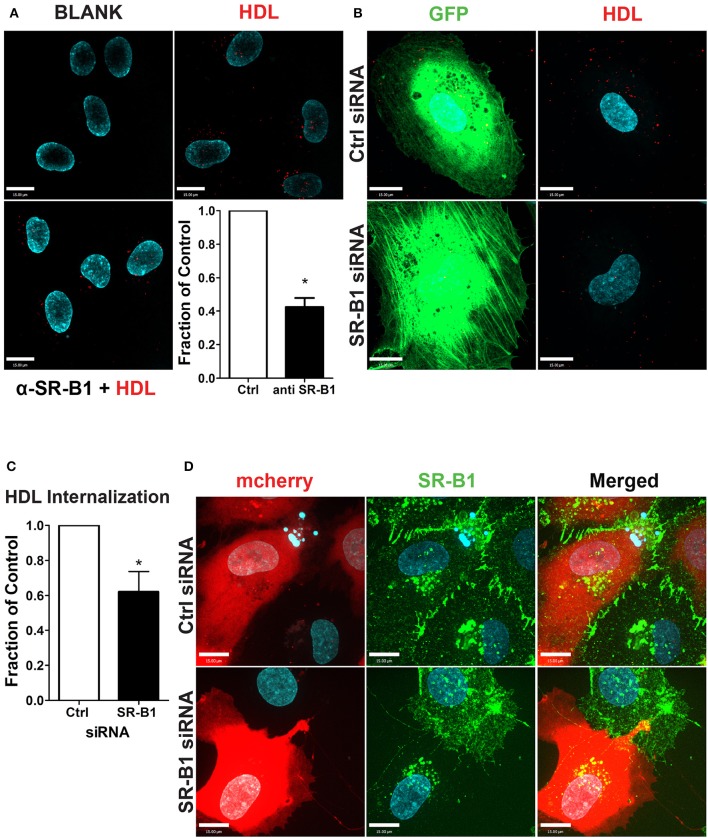
SR-BI is required for HDL internalization. **(A)** The addition of unlabeled anti-SR-BI nanobody which binds to the extracellular domain of SR-BI partially blocks HDL internalization. *n* = 3; approximately 40 cells were observed for each condition. **(B)** HCCMECs were co-transfected (molar ratio 1:100) with GFP and siRNA against SR-BI or a non-targeting control (Ctrl) to test the effect of SR-BI on HDL internalization. **(C)** 48 h post transfection, SR-BI siRNA transfected cells showed a decrease in HDL internalization. *n* = 5; approximately 25 transfected cells were observed for each condition. Scale Bars = 15 μm. ^*^p < 0.01. **(D)** Effective knockdown of SR-BI in co-transfected cells was verified by immunofluorescence (SR-BI in green; mCherry in red).

To test the functional role of SR-BI in HDL internalization, HCCMECs were pretreated with a blocking scFv to SR-BI which decreased AF568-HDL internalization by ≈57% (Figure 5A) while depletion of SR-BI by siRNA (assessed through immunofluorescence, see Figure 5D) significantly attenuated HDL internalization by ≈40% (Figures 5B,C). Together, these experiments strongly implicate SR-BI in the internalization of HDL by primary cerebral microvascular endothelial cells in a clathrin- and caveolae-independent manner.

### The SR-BI scaffolding protein, PDZK1, is not required for HDL internalization

**Figure 6:**
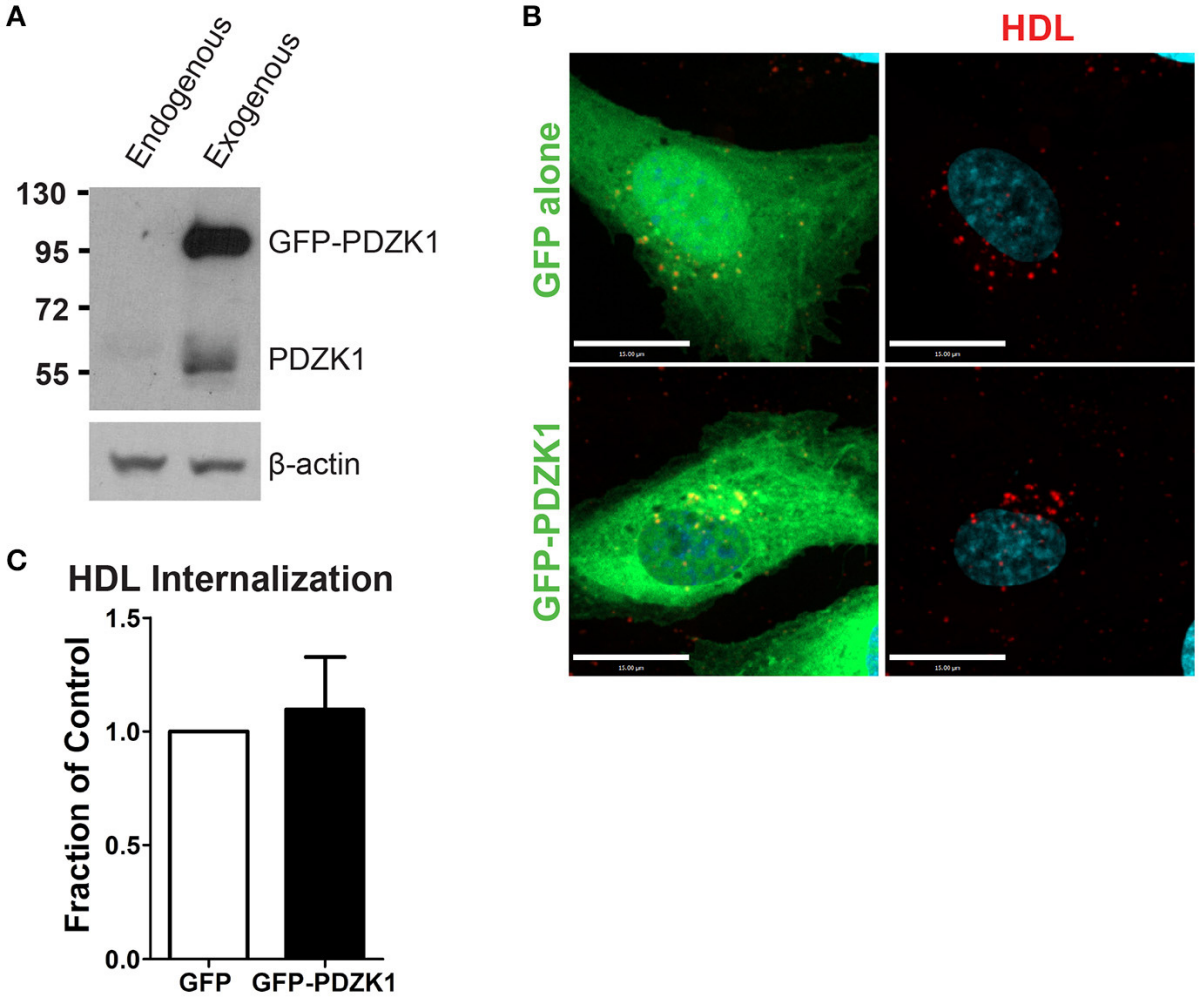
PDZK1 is not required for HDL internalization in HCCMECs. **(A)** Western blot showing the expression of endogenous PDZK1 in HCCMECs compared to positive control, HCCMECs overexpressing PDZK1. **(B)** 48 h post transfection of GFP alone or GFP-PDZK1, DiI-HDL was internalized for 10 min which showed that over-expressed GFP-PDZK1 had no significant effect on DiI-HDL internalization. Quantification can be found in **(C)**; *n* = 5; approximately 25 transfected cells were observed for each condition. Scale Bars = 15 μm.

In the liver, the adaptor protein PDZ-containing 1 (PDZK1) is essential for the stability of SR-BI at the plasma membrane (Kocher et al., 2003). Furthermore, in bovine aortic endothelial cells, HDL binding to SR-BI stimulates activation of endothelial nitric oxide synthase and NO production in a PDZK1-dependent manner (Zhu et al., 2008). To determine whether PDZK1 is also involved in HDL internalization upon its binding to SR-BI in brain microvascular endothelial cells, the expression levels of PDZK1 were assessed by Western blot analysis. HCCMEC expressed almost undetectable levels of PDZK1 (Figure 6A); this was supported by qPCR demonstrating essentially no PDZK1 mRNA in HCCMEC (data not shown). This suggests that PDZK1 is very unlikely to play a major role in HDL uptake or transcytosis in HCCMECs. In support of this notion, overexpression of PDZK1 in HCCMECs had no significant impact on DiI-HDL internalization (Figures 6B,C).

### Endothelial nitric oxide production is not required for HDL internalization

**Figure 7:**
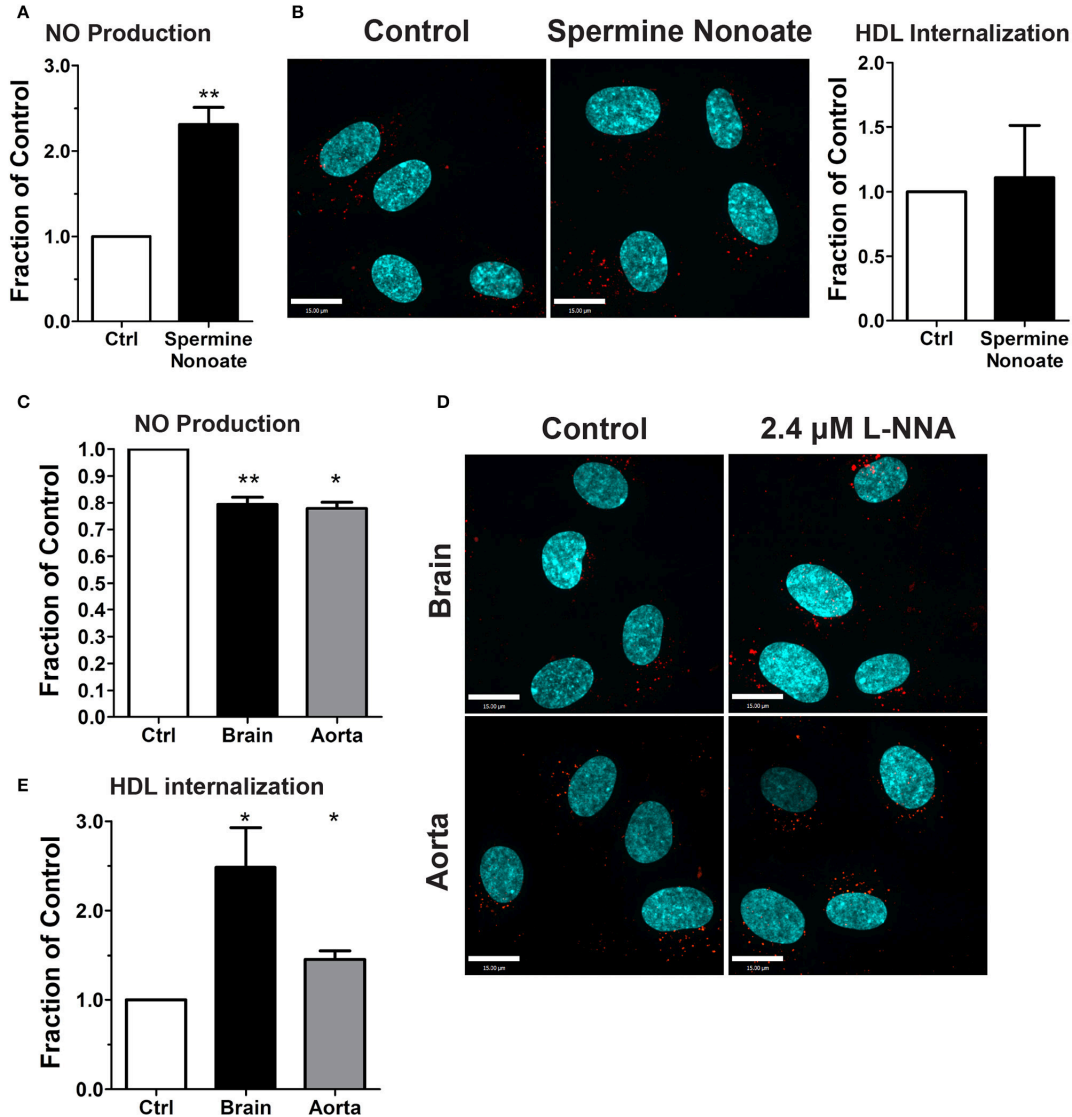
Inhibition of nitric oxide synthase does not affect HDL internalization by HCCMECs. **(A)** The NO donor spermine nonoate significantly increased DAF fluorescence, validating this NO-sensor in HCCMEC. *n* = 5; 16 random fields per treatment for each *n* and each image had approximately 100 cells. **(B)** Spermine nonoate did not affect HDL internalization. *n* = 5; at least 40 cells were examined per condition per experiment. **(C)** The nitric oxide synthase inhibitor L-NNA reduced basal NO levels in HCCMECs and HAECs as measured by DAF-FM (HCCMECs, *n* = 5; HAECs, *n* = 3. ^**^p < 0.005, ^*^p < 0.05), **(D)** L-NNA significantly increased HDL internalization by approximately 2.5-fold in HCCMECs but only by 1.4- fold in HAECs, quantified in **(E)** (HCCMECs, *n* = 5; HAECs, *n* = 3. ^*^p < 0.05). Scale Bars = 15 μm.

The dispensability of PDZK1 for HDL uptake and transcytosis brought into question the role of downstream eNOS activation and nitric oxide (NO) production. The release of exogenous NO using the donor spermine NONOate - which was verified by using the sensitive intracellular NO sensor 4-Amino-5-Methylamino-2′,7′-Difluorofluorescein (DAF-FM) Diacetate (Figure 7A; Nakatsubo et al., 1998) - had no effect on HDL internalization (Figure 7B). Next, basal NO production was significantly reduced by the classic eNOS inhibitor L-NNA (Figure 7C); this led to an increase in AF568-HDL internalization by ≈2.5-fold (Figures 7D,E). L-NNA reduced basal NO production in human aortic endothelial cells to a similar degree as in the brain endothelial cells (Figure 7C), but this increased AF568-HDL internalization by only ≈40% (Figures 7D,E). These results suggest that there may be different mechanisms involved in HDL internalization by different vascular beds. Future studies will be needed to better understand the regulation post-endocytosis of the receptor and the fate of the ligand.

## Discussion

Using a combination of high-resolution microscopy techniques, we have observed that HDL internalization by cerebral cortex microvascular endothelial cells is performed by SR-BI in a manner that is independent of clathrin or caveolin-1 (Figures 2E, 5) yet requires dynamin and cholesterol (Figures 2C,D). This suggests that other modes of endocytosis are responsible for endocytosis of SR-BI and the initiation of transcytosis in these cells. Two potential endocytic portals that are sensitive to cholesterol perturbations are flotilin-mediated endocytosis (Aït-Slimane et al., 2009) or the Cdc42-mediated clathrin-independent carrier (CLIC)/Glycosylphosphatidylinositol-linked proteins enriched compartments (GEEC) (Chadda et al., 2007). To our knowledge these types of endocytosis have not been implicated in the uptake of SR-BI although most of these studies have been conducted in hepatocytes, macrophages or other endothelial cell types.

In contrast to the canonical SR-BI signaling pathway employed by hepatocytes and aortic endothelial cells, (Saddar et al., 2010) PDZK1 and nitric oxide are not required for HDL internalization in the brain (Figures 6, 7). A strength of our approach is the ability to distinguish transcytosis from the paracellular permeability of endothelial monolayers, a problem that confounds experiments with traditional transwells particularly when applying chemical inhibitors or attempting molecular manipulation by transfection (Armstrong et al., 2012).

While we are not able to achieve complete knock-down of SR-BI, our data indicate that SR-BI is likely not the sole receptor responsible for HDL internalization and transcytosis across the BBB (Figure 5). Other candidates include the adenosine triphosphate (ATP)-binding cassette transporters ABCG1(Rohrer et al., 2009) and the β-chain of cell surface F_0_F_1_ ATPase (Cavelier et al., 2012) which have been reported to perform HDL transcytosis in bovine aortic endothelium. We were unsuccessful in depleting endothelial cells of ABCG1 due to the resistance of endothelial cells to transfection. Additionally, while our study has focused on the trans-endothelial transport of the entire HDL particle, it is important to note that the transcytosis of HDL and its principal apolipoprotein (ApoA1) can be regulated differently. For instance, studies in bovine aortic endothelium have indicated that HDL transcytosis is mediated by receptors ABCG1 and SR-BI, while ApoA1 transcytosis has been reported to involve the receptor ABCA1. In our experiments, HDL internalization was almost entirely inhibited by the addition of recombinant ApoA1 but reduced by about 50% in the absence of SR-BI; this suggests that ABCG1 may play a significant role in HDL transcytosis by the brain microvascular endothelium.

We observed that the effect of inhibiting eNOS on HDL internalization was more severe in the brain compared to the aorta suggesting that there may also be tissue-specific mechanisms of HDL internalization. This is not surprising given the functional and structural differences between the macro- and micro-vasculature (Aird, 2007a,b, 2012). Without knowing more about the precise regulation of HDL uptake in the brain endothelial cells it is difficult to predict the targets of NO. A recent study found that NO promoted insulin transcytosis by inactivating protein tyrosine phosphatase 1B (Wang et al., 2013). One possibility is that in the HCCMECs in the absence of NO, clathrin-mediated endocytosis is attenuated and this stimulates the endocytic portal responsible for SR-BI uptake. Tissue specificity was also evident from our finding that SR-BI is in caveolin1-deficient sub-fractions of brain endothelial lysates, while SR-BI has been previously reported in the caveolin1-rich sub-fractions (Uittenbogaard et al., 2000) and we have confirmed this in human coronary endothelial cells (not published). Indeed, the expression of Mfsd2a—the lysophospholipid transporter and *de facto* inhibitor of caveola—appears to be restricted to brain endothelial cells.

After internalization, the HDL particle may face several fates including recycling to the apical surface, endocytic maturation and degradation, and transcytosis to the basal membrane. The lack of a simple system to study HDL transcytosis has limited our understanding of its intracellular fate, including whether the pH of the internalized vesicle changes during transcytosis. Furthermore, the relationship of transcytosis to the cellular cytoskeleton and microtubule network remains undefined. For example, while it is reasonable to speculate that transcytotic vesicles use microtubule-based motors to transit from the apical to the basal membrane (Bomsel et al., 1990), this has not yet been established in the endothelium. It is our hope that the fast live-cell imaging techniques including our TIRF assay for HDL transcytosis will enable rapid progress in answering these questions.

Finally, while our study has focused on the cerebral microcirculation, HDL transcytosis also occurs in the systemic circulation where it is postulated to play a role in the return of excess cholesterol from the arterial wall to the liver (i.e., reverse cholesterol transport) (Phillips, 2014). In this process, circulating HDL is believed to enter the arterial intima by endothelial transcytosis and then into the lymphatics prior to returning to the liver. Whether a similar process occurs in the brain is unknown, although HDL-like particles have been observed in cerebrospinal fluid (Koch et al., 2001). The high degree of tightness of cell-cell junctions in the blood-brain barrier compared to the systemic circulation may indicate that transcytosis is likely to make a relatively larger contribution in the CNS to basal endothelial permeability. Combined with the fact that ApoA1is not synthesized in the CNS and that higher tissue levels of ApoA1 may be protective against CNS disease, (Kontush and Chapman, 2008) the ability to enhance HDL and ApoA1 transcytosis across the cerebral microcirculation may provide significant therapeutic opportunities; this will necessitate a greater understanding of its regulation. Accordingly, our data hint at signaling pathways downstream of SR-BI that may be unique to the cerebral microcirculation; in particular, the mechanisms by which inhibition of eNOS apparently stimulates HDL internalization but not transcytosis is unknown and will require further investigation.

## Conclusion

**Figure 8:**
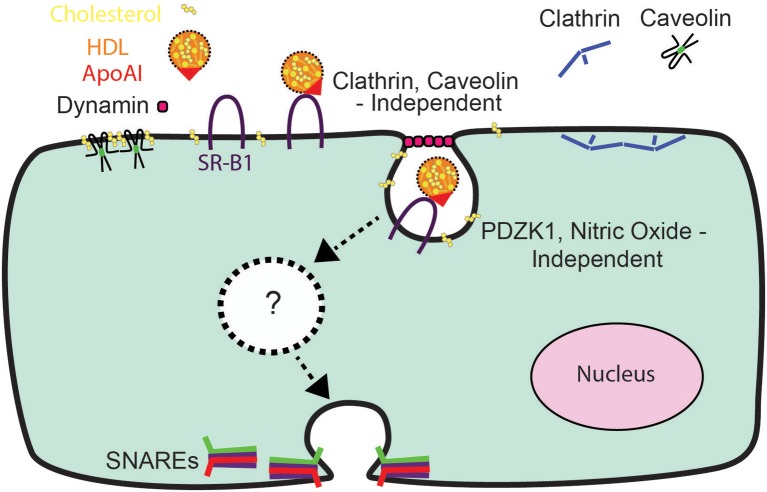
A Schematic representation of HDL transcytosis by HCCMECs.

In conclusion, the relative impermeability of the blood-brain barrier and the high prevalence of diseases affecting the brain (Qiu et al., 2009) have heightened interest in understanding the mechanisms of HDL transcytosis. Our data indicate that HDL transcytosis is regulated in a tissue-specific manner, with SR-BI displaying non-canonical signaling by cerebral cortex endothelial cells (see Figure 8). While we were unable to identify the precise dynamin-dependent endocytic carrier our results show that HDL and SR-BI are internalized and transit from the apical surface to the basolateral. Future studies will focus on determining the nature of the carrier and whether the carrier transits directly, or undergoes fission and fusion events with endosomes or other vesicles while in transit. We anticipate that the single-cell assay for HDL transcytosis described herein may facilitate the elucidation of its molecular regulation.

## Author contributions

KF conducted experiments, analyzed data, assembled figures and wrote a draft of the manuscript. CW conducted experiments and analyzed data. SN generated novel reagents and assisted with experiments. BH analyzed the data. GF and WL conceived the study and wrote the manuscript.

### Conflict of interest statement

The authors declare that the research was conducted in the absence of any commercial or financial relationships that could be construed as a potential conflict of interest.
