# Supplementary material for: SR-BI Mediated Transcytosis of HDL in Brain Microvascular Endothelial Cells Is Independent of Caveolin, Clathrin, and PDZK1
Source: Front Physiol. 2017 Oct 30;8:841. doi: 10.3389/fphys.2017.00841 (PMC5670330; doi:10.3389/fphys.2017.00841)
Supplement: Supplementary file 1 [file DataSheet1.DOCX]

Supplementary Material

Transcytosis of HDL in brain microvascular endothelial cells by SR-BI is independent of clathrin and PDZK1

Karen Y.Y. Fung^1,2^, Changsen Wang^2^, Steffen Nyegaard^3^, Bryan Heit^4^, Gregory D. Fairn*^1,2,5^ and Warren L. Lee*^1,2,6^

^1^Department of Biochemistry, University of Toronto, Toronto, ON, Canada

^2^Keenan Research Center for Biomedical Science, St. Michael’s Hospital, Toronto, ON, Canada

^3^Program in Cell Biology, Hospital for Sick Children, Toronto, ON, Canada.

^4^Department of Microbiology and Immunology and the Centre for Human Immunology, the University of Western Ontario, London, ON, Canada.

^5^Department of Surgery, University of Toronto, ON, Canada.

^6^Departments of Medicine and Laboratory Medicine and Pathobiology, University of Toronto, ON, Canada.

*Co-Corresponding authors: [Leew@smh.ca](mailto:Leew@smh.ca) and [FairnG@smh.ca](mailto:FairnG@smh.ca)

St. Michael’s Hospital

Toronto, Ontario, CANADA

M5B 1W8

# Supplementary Figures

**Supplemental Figure 1. Expression and purification of recombinant ApoA1.** The process of recombinant ApoA1 purification from cobalt resin as analyzed by SDS-PAGE and staining with coomassie blue. The elution contains ApoA1 only and lacks any contaminating protein.
