# Supplementary figures and images for: SR-BI Mediated Transcytosis of HDL in Brain Microvascular Endothelial Cells Is Independent of Caveolin, Clathrin, and PDZK1
Source: Front Physiol. 2017 Oct 30;8:841. doi: 10.3389/fphys.2017.00841 (PMC5670330; doi:10.3389/fphys.2017.00841)

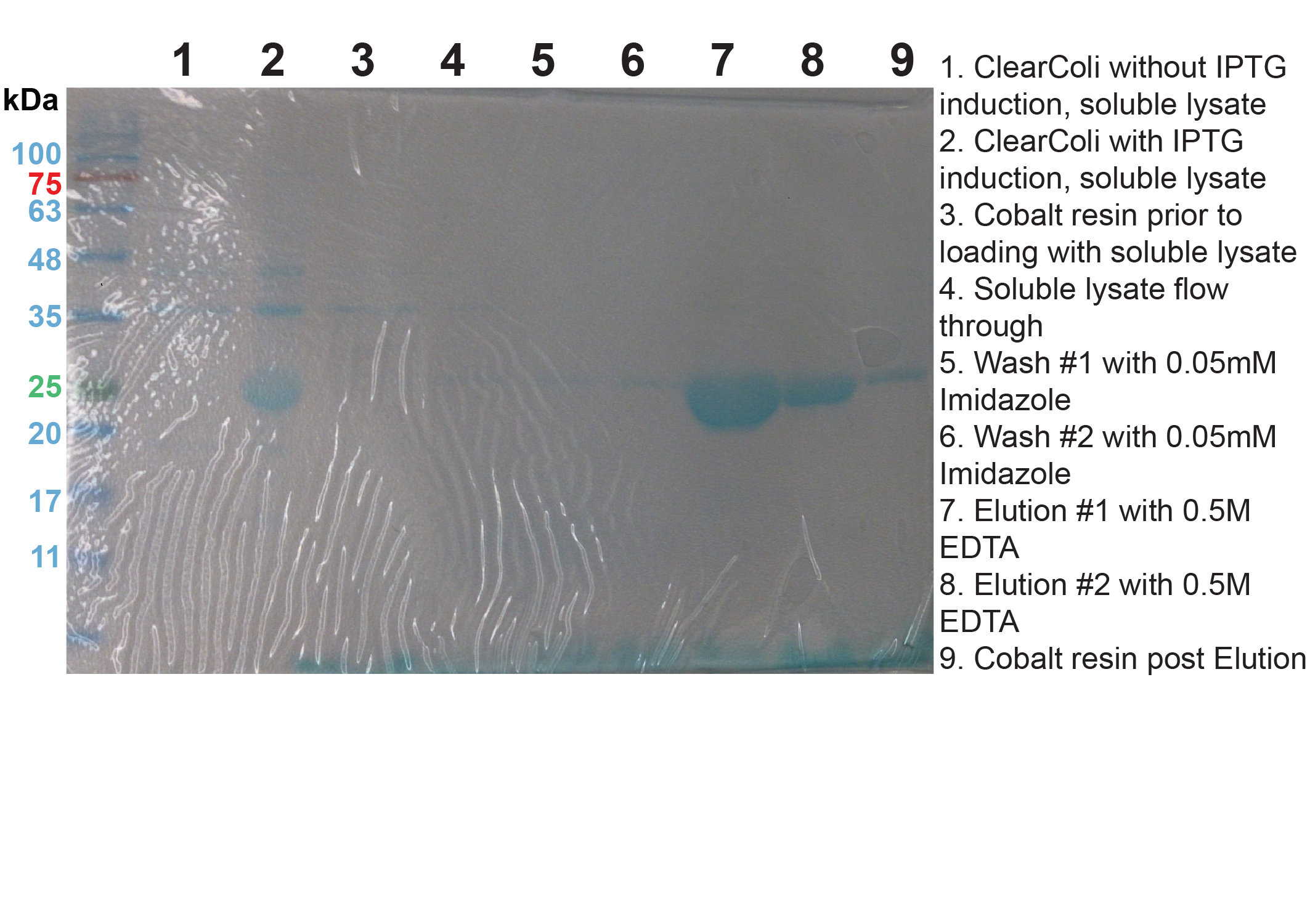

Supplement: Supplementary file 2 [file Image1.TIF]
